# Haematological reference intervals for pregnant Icelandic mares on pasture

**DOI:** 10.1186/s13028-023-00721-x

**Published:** 2023-12-15

**Authors:** Charlotta Oddsdóttir, Hanna Kristrún Jónsdóttir, Erla Sturludóttir

**Affiliations:** 1 Division of Bacteriology and Pathology, Department of Pathology, Institute for Experimental Pathology at Keldur, Keldnavegi 3, 112 Reykjavík, Iceland; 2 Faculty of Agricultural Sciences, Agricultural University of Iceland, Árleyni 22, 112 Reykjavík, Iceland

**Keywords:** Erythrocytes, Horses, IDEXX Catalyst one, IDEXX ProCyte, Lactation, Leukocytes, Pregnancy, Reference population

## Abstract

**Background:**

Few studies have been conducted on haematological reference intervals (RIs) in Icelandic horses. Reference intervals have been published for Icelandic horses in Austria and a preliminary study in Iceland compared haematological values in riding horses to published RIs for other breeds as well as Icelandic horses abroad. Haematological parameters can vary greatly due to factors such as breed, gender, age, reproductive status, and training, as well as feeding, prior exercise and management method. Icelandic broodmares are kept on pasture under supervision throughout the year, with haylage provided during the winter, and it is therefore of interest to establish haematological reference intervals for pregnant broodmares in Iceland. The purpose of this study was to establish haematological RIs specific to Icelandic broodmares in the first months of pregnancy, kept on pasture. Blood samples from 183 mares, stabilised in EDTA were analysed using IDEXX ProCyte Dx and total protein was analysed in serum samples from 157 of the 183 mares, using IDEXX Catalyst One analyser. The RIs were established using the guidelines of the American Society for Veterinary Clinical Pathology.

**Results:**

The RIs for red blood cell count, haematocrit and haemoglobin were higher in pasture-kept Icelandic mares in early pregnancy, most of which were lactating, than in pregnant mares of other breeds. This was also true for white blood cell count, as well as numbers of monocytes, eosinophils, and basophils, which in some instances might illustrate problems in the automated categorisation of some leukocytes.

**Conclusions:**

As no RIs have been published for other pasture-kept Icelandic horses, future investigations should include other groups of pasture-kept Icelandic horses. Such an analysis might elucidate the effect of breed, management, and pregnancy on haematological values in pasture-kept Icelandic horses.

## Background

Few studies have been conducted on haematological reference intervals (RIs) in Icelandic horses. Reference intervals have been published for Icelandic horses in Austria [1] indicating some differences in their haemogram when compared to general RIs for horses. In a preliminary study, it was found that haemoglobin (Hb) and haematocrit (Hct) values were lower in 78 riding horses in Iceland than published RIs for other breeds as well as Icelandic horses abroad [2]. Haematological parameters can be influenced by a variety of factors, such as breed, gender, age, reproductive status, and training, as well as feeding and prior exercise [3–6]. Furthermore, certain haematological values can vary considerably between individual horses [7]. For these reasons it is important to establish reference intervals for haematological values in pregnant Icelandic mares to be able to distinguish physiological changes from pathological conditions. Such RIs will also be a valuable tool when assessing the welfare of mares being harvested for blood for eCG production during this gestational period. Icelandic broodmares are kept on pasture under supervision throughout the year, with haylage provided during the winter. Management method and feeding can influence physiology and haematology [8] and it is therefore of interest to establish haematological reference intervals for pregnant broodmares on pasture in Iceland.

The purpose of this study was to establish haematological RIs specific to Icelandic broodmares in the first four months of pregnancy that are kept on pasture.

## Methods

### Animals

The study population consisted of four groups of broodmares, all registered in the Worldfengur studbook of the Icelandic Horse (www.worldfengur.com). In the Northwest of Iceland, two herds of mares were sampled: 62 mares (5–21 years old) and 11 mares (4–22 years old). In the South of Iceland, two herds of mares were sampled: 97 mares (4–22 years old) and 13 mares (5–19 years old). Mares were kept free-range in open grazing areas with unlimited access to fresh water and brought in along with their foals into a corral for blood sampling, between 9:00 and 13:00. They were all non-fasted, in the first trimester of pregnancy and 162 of them (86%) were lactating. Experienced farm personnel handled all mares to ensure animal safety and minimise stress.

### Samples

Blood samples were collected over eight weeks in July-September 2022. Blood was taken from the jugular vein after overlying skin had been clipped and disinfected and collected into 2 mL EDTA tubes (Vacuette, Greiner Bio-One, Austria) for haematological analysis, and 4 mL serum tubes with a clot activator and separator gel (Vacuette, Greiner Bio-One, Austria) for the analysis of serum total protein. Blood tubes were turned upside down several times, labelled with the ID number of each mare and kept upright at 4 °C overnight, until analysis was done the following day. A maximum of 30 h elapsed from sampling until haematological analysis was done. Serum tubes were allowed to clot, centrifuged at 1932 x g for 10 min at 4 °C, for serum to be harvested and frozen at -20 °C until analysed.

### Haematological and total protein analysis

Haematological values were assessed in whole EDTA stabilised blood samples by an IDEXX ProCyte Dx analyser (IDEXX Laboratories, Inc., ME, USA), employing equine-specific, as well as breed and sex specific software settings. Before analysis samples were allowed to reach room temperature (~ 20 °C). The following values were analysed: red blood cell count (RBC), haematocrit (Hct), haemoglobin (Hgb), mean corpuscular volume (MCV), mean corpuscular haemoglobin concentration (MCHC), mean corpuscular haemoglobin (MCH), red cell distribution width (RDW), white blood cell count (WBC), as well as numbers of neutrophils (NEU), lymphocytes (LYM), monocytes (MONO), eosinophils (EOS) and basophils (BASO). Visual differential count and assessment of platelet aggregation were done on blood smears in the case of abnormally high or low platelet counts, as well as if the analyser gave an indication of abnormal leukocyte counts.

Total protein (TP) values were measured in serum using an IDEXX Catalyst One (IDEXX Laboratories, Inc., ME, USA), based on the biuret method [9], excluding haemolyzed samples to reduce the risk of falsely increased values [10].

### Statistics

The resulting values were used to determine RIs for haematological values and serum total protein values. Guidelines from the American Society for Veterinary Clinical Pathology [11] were followed to estimate reference intervals. The haematological values EOS and BASO were log-transformed to achieve normal distribution. The Rosner test [12] was used to detect outliers and the outliers were removed prior to finding the 2.5th and the 97.5th percentiles which determines the lower and upper reference values, respectively. Bootstrapping was used to estimate the 90% confidence interval for the upper and lower reference values. All statistical analysis was done in R [13].

## Results

**Fig. 1:**
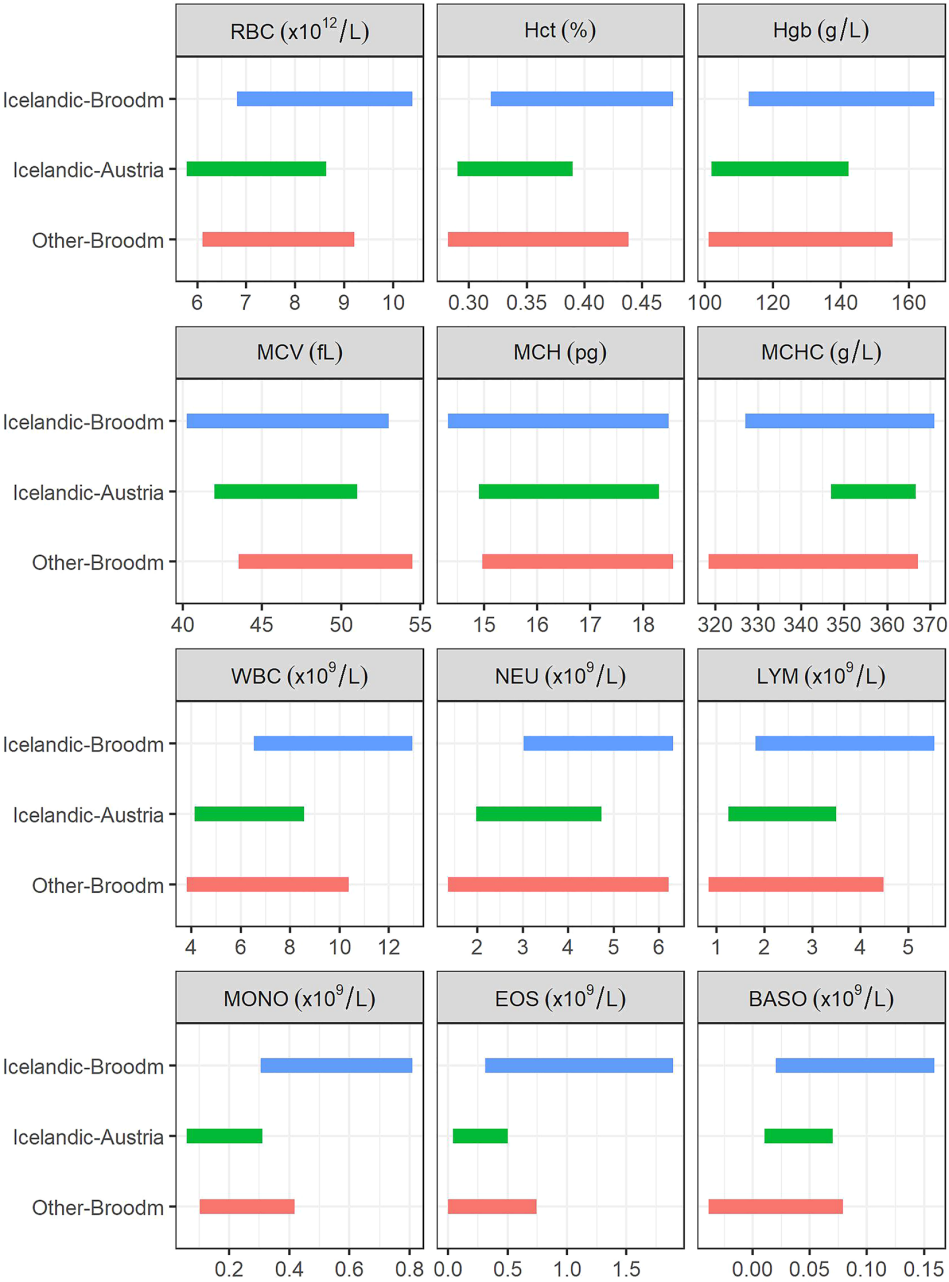
Reference intervals for haematological values from Icelandic broodmares, non-pregnant Icelandic horses in Austria and broodmares from other breeds. Reference intervals (RIs) for haematological values from Icelandic broodmares (Icelandic-Broodm) in this study compared to RIs for Icelandic horses in Austria (Icelandic-Austria) [1], and for broodmares of other breeds (Other-Broodm) [5] as calculated from the means and* with *an assumption of mean ± 1.96*SD. *RBC *red blood cell count,* Hct *haematocrit,* Hgb *haemoglobin,* MCV *mean corpuscular volume,* MCH *mean corpuscular haemoglobin,* MCHC *mean corpuscular haemoglobin concentration,* WBC *white blood cell count,* NEU *neutrophil count,* LYM *lymphocyte count,* MONO *monocyte count,* EOS *eosinophil count,* BASO *basophil count

All mares remained healthy and clinically free of disease during the study period. Haematological values were analysed in 183 samples and total protein in 157 serum samples, see Table 1. Single values for Hct, WBC, LYM, MONO and BASO were classified as outliers and removed from the data before the RIs were estimated. Serum samples for TP analysis from 166 mares were available, out of which nine were not analysed due to haemolysis. Table 2 shows the RIs calculated from the haematological values. Figure 1 shows a graphic comparison between our RIs for pregnant Icelandic broodmares and RIs for Icelandic horses residing in Austria [1] and pregnant mares of other breeds residing in California [5]. Platelet count in one mare was abnormally low and visual inspection of a blood smear showed platelet aggregation, confirming pseudothrombocytopenia, but as platelet values were not included in the statistical analysis, this had no effect on the results. Automated analysis of one blood sample indicated the presence of band neutrophils, but this was disproved by visual inspection of a blood smear, and as the manual differential count did not differ from the automated one, the automated analysis results from this mare were included.

**Table 1:** Descriptive statistics for haematological values, serum total protein and age for Icelandic broodmares and outliers that were removed from the data

Analyte	Unit	N	Min	Mean	SD	Median	Max	Outlier
RBC	x10^12^/L	183	5.83	8.40	0.982	8.31	11.44	
Hct	%	182	26.2	38.9	4.4	38.8	52.5	56.4
Hgb	g/L	183	99	137	13.7	137	185	
MCV	fL	183	37.5	46.6	3.354	46.5	55.2	
MCH	pg	183	13.5	16.4	1.078	16.5	19.2	
MCHC	g/L	183	320	353	11.15	354	378	
RDW	%	183	26.1	30.8	2.22	30.7	37.4	
WBC	x10^9^/L	182	5.21	9.42	1.656	9.26	13.72	16.22
NEU	x10^9^/L	183	1.80	4.47	0.861	4.41	7.27	
LYM	x10^9^/L	182	1.48	3.51	1.005	3.35	7.05	7.79
MONO	x10^9^/L	182	0.27	0.56	0.125	0.56	0.85	1.11
EOS	x10^9^/L	183	0.18	0.82	0.440	0.74	3.13	
BASO	x10^9^/L	182	0.01	0.07	0.039	0.06	0.23	0.71
TP	g/L	157	58	71.8	5.5	72	85	
Age	years	183	4.0	11.3	4.4	11.0	22.0	

Blood samples from pregnant Icelandic broodmares kept on pasture were analysed using IDEXX ProCyte Dx analyser (haematological values) and IDEXX Catalyst One analyser (serum total protein values). Serum samples from 166 mares were available, out of which nine were not analysed due to haemolysis. Five haematological values were outliers and therefore removed from the data

*RBC* red blood cell count,* Hct* haematocrit,* Hgb* haemoglobin,* MCV* mean corpuscular volume,* MCH* mean corpuscular haemoglobin,* MCHC* mean corpuscular haemoglobin concentration,* RDW* red cell distribution width,* WBC* white blood cell count,* NEU* neutrophil count,* LYM* lymphocyte count,* MONO* monocyte count,* EOS* eosinophil count,* BASO* basophil count,* TP* serum total protein

**Table 2:** Reference interval (RI) for haematological values and serum total protein for pregnant Icelandic broodmares

Analyte	unit	RI	LRI90%	URI90%
RBC	x10^12^/L	6.81–10.39	6.49–6.96	10.03–10.96
Hct	%	31.9–47.7	30.8–32.6	47–48.7
Hgb	g/L	113–167	111–117	161–169
MCV	fL	40.3–53	39.8–41	52.2–54.6
MCH	pg	14.3–18.5	14–14.7	18–18.8
MCHC	g/L	327–371	326–333	368–374
RDW	%	27.1–35.4	26.5–27.3	34.7–37.3
WBC	x10^9^/L	6.53–12.95	6.20-7.00	12.32–13.55
NEU	x10^9^/L	3.02–6.31	2.73–3.15	6.07–6.58
LYM	x10^9^/L	1.81–5.54	1.69–2.16	5.22–6.9
MONO	x10^9^/L	0.3–0.81	0.29–0.36	0.76–0.83
EOS	x10^9^/L	0.31–1.89	0.26–0.34	1.62–2.04
BASO	x10^9^/L	0.02–0.16	0.01–0.02	0.13–0.22
TP	g/L	62.0–81.1	59.0–63.0	80.1–83.0

Reference intervals (RIs) for haematological values and serum total protein were calculated for pregnant Icelandic broodmares on pasture.

*RBC* red blood cell count,* Hct* haematocrit,* Hgb* haemoglobin,* MCV* mean corpuscular volume,* MCH* mean corpuscular haemoglobin,* MCHC* mean corpuscular haemoglobin concentration,* RDW* red cell distribution width,* WBC* white blood cell count,* NEU* neutrophil count,* LYM* lymphocyte count,* MONO* monocyte count,* EOS* eosinophil count,* BASO* basophil count,* TP* serum total protein,* LRI90%* 90% confidence interval for lower reference values,* URI90%* 90% confidence interval for upper reference values

## Discussion

When comparing reference intervals and values between subpopulations and between different studies, it should be borne in mind that methodology, equipment, stress levels and other factors can make comparison difficult. In the present study, the apparatus used to analyse samples is the most widely used by veterinary practices in Iceland and the resulting RIs will therefore be useful to Icelandic equine practitioners. Also, the methods used to sample, store and measure blood will invariably influence results. In this study, samples needed to be transported to the laboratory before analysing and to ensure uniformity, the analysis was carried out with the same delay for all samples, even if some were received earlier. During transport, samples were kept cool, so that temperature fluctuations were avoided between batches. The use of EDTA, refrigeration and delay of analysis of blood samples is known to lead to altered estimates of platelets [14], and this was the case in our material. Visual assessment of the one sample showing low platelet count confirmed pseudothrombocytopenia and as it was known that platelet count could be unreliable using the sampling methods chosen, it was decided to exclude all values for platelets in this study. It must also be pointed out that in a clinical setting, the methods of blood sampling will often involve EDTA, delay and temperature fluctuations, and if platelet analysis was specifically needed, more appropriate sampling methods would be advised.

The only published study on haematological RIs in Icelandic horses was conducted on riding horses in Austria, predominantly geldings [1], and a comparison with our study population should therefore be made with caution. In establishing RIs, the confidence interval should not exceed 0.2 times the RI [11]. This was exceeded in the case of the upper reference limit for RBC, RDW, LYM and for the lower value for MCHC, EOS and BASO. The uncertainty can therefore be considered high for those RIs. However, it should also be noted that the number of mares in our study is considerably higher than in many previous studies [4, 5, 15] making our RIs statistically well-grounded. The upper limits of RIs for RBC, Hct and Hgb, as well as WBC, in Icelandic broodmares were higher than in Icelandic horses in Austria and broodmares of other breeds [1, 5]. Results of other studies on pregnant mares were less suitable for calculation of RIs and therefore not included in the Figs. [16, 17], however the means for RBC, Hct and Hgb were lower than in our study. It is quite possible that management has some influence on these results [8]. Icelandic broodmares are generally kept under extensive management (free-range at pasture, with haylage provided in winter) as was the case for the herds sampled in this study. The study population is therefore representative of most broodmares in Iceland. Broodmares in other studies were kept under various management systems, including individual management by their owners [5], homologous feeding and management at a research station [16] or free grazing [17] as were the mares in the present study. The mares in our study were kept on large pastures with varying grazing, giving them nutritional variety, which can account for some differences when comparing these studies, in addition to the effect of breed and methodology.

The mares in the present study exhibited a relatively large variation in erythrocyte size, with a mean RDW value of 30.8%, even though MCV was lower than most reports of this stage in equine pregnancy [5, 15, 16]. In humans, a decreased lifespan of erythrocytes can lead to an increase in the variation of MCV, leading to an increased RDW during late gestation [18]. Studies on mares of various breeds have however indicated that MCV does not change from the non-pregnant state to the first trimester [5, 16] or even, in the case of Breton mares, can be reduced during this period [17]. The mares in the present study were in the first trimester, but the RI (27.1–35.4%) for RDW in this study was higher than the ProCyte Dx equine RI of 24.6–33.,3%. In stabled Icelandic horses in Austria, the RI for RDW was found to be 16.8–19.6 [1], but in that study, the standard deviation or coefficient of variation are not presented and comparison to our results is therefore not possible. It can be difficult to compare RDW values between publications, as this parameter is often not presented [5, 13, 15–17], and values obtained using different analysers are not always compatible [19]. In our laboratory, RDW values in Icelandic horses are generally higher than found in the study by Leidinger et al. [1], regardless of sex, health or reproductive status.

The mares were in the first trimester of pregnancy and most of them were nursing 2–4-month-old foals. Our findings on erythrocyte values such as Hct and MCV are in line with previous findings in lactating mares in the first 4 months after foaling, although it is not clear if those mares were also pregnant [20]. Also, information on lactation status in the literature on broodmare haematology is sometimes lacking [5]. In the present study, when lactating and non-lactating broodmares (n = 21) were compared it was noticed that the non-lactating broodmares had a narrower range in haematological values. Therefore, the RI may not be appropriate for non-lactating, pregnant broodmares even though this might purely reflect the small number of non-lactating mares. In our study, the resulting RI for RBC, Hct, Hb and TP were higher than for pregnant mares in a previous study [5], and this could indicate a haemoconcentration, either due to truly higher erythrocyte numbers or due to dehydration. As the mares in our study had good access to fresh water before and immediately after being brought to the corral for blood collection, herd-wide dehydration is unlikely. When it comes to the mean values of RBC, Hct and Hgb, generally they are above average in the Icelandic mares, compared to the mean values found in other breeds in the first trimester, with similar MCV, MCH and MCHC values between breeds [5, 15–17]. At each sampling, care was taken to cause minimal stress when bringing mares into the corral, with the same method used each time to reduce the effect of stress on haemoconcentration, which is known to occur following splenic contraction in horses [21]. TP was analysed to distinguish between high Hct values due to splenic contraction and increase in erythrocyte production [22]. The highest TP values measured in the present study were comparable to previous results on broodmares of other breeds [23].

Slightly higher WBC values were found in our study than in studies of other breeds [5, 16, 17], mostly explained by higher numbers of eosinophils and monocytes, even though one study did find even higher WBC values in Carthusian mares [15]. It is possible that in some cases, lymphocytes could have been wrongly classified as monocytes [24], but it should also be considered that this would not have an influence on total numbers of leukocytes. It has been postulated that horses kept on pasture exhibit a rise in eosinophils during the late summer and autumn due to increased presence of gastrointestinal nematodes [25], although one study concluded that significant increase in eosinophil numbers in pregnant mares were most likely related to pregnancy [5].

## Conclusions

This study has yielded RIs for pasture-kept Icelandic mares in early pregnancy, most of which were nursing 2-4-month-old foals. These mares exhibited higher RIs for red blood cell count, haematocrit and haemoglobin when compared to pregnant mares of other breeds and to Icelandic riding horses in Austria. The same was true for numbers of leukocytes, as well as numbers of monocytes, eosinophils, and basophils, which in some cases might come from difficulties in automated categorisation of some leukocytes. As no RIs have been published for pasture-kept non-pregnant mares in Iceland, nor any other pasture-kept Icelandic horses, it is not possible to draw conclusions as to the influence of pregnancy on haematological values. This illustrates the need for further investigations in the future.

## Data Availability

Not applicable.

## Declarations

### Prior publication

Data have not been published previously.
